# Ectopic Expression of MiR-125a Inhibits the Proliferation and Metastasis of Hepatocellular Carcinoma by Targeting MMP11 and VEGF

**DOI:** 10.1371/journal.pone.0040169

**Published:** 2012-06-29

**Authors:** Qian Bi, Shanhong Tang, Lin Xia, Rui Du, Rui Fan, Liucun Gao, Jiang Jin, Shuhui Liang, Zheng Chen, Guanghui Xu, Yongzhan Nie, Kaichun Wu, Jie Liu, Yongquan Shi, Jie Ding, Daiming Fan

**Affiliations:** 1 State Key Laboratory of Cancer Biology, Xijing Hospital of Digestive Diseases, Fourth Military Medical University, Xi’an, P.R. China; 2 Department of Digestive Diseases, Huashan Hospital, Fudan University, Shanghai, China; University of Hong Kong, Hong Kong

## Abstract

**Background:**

Studies have been shown that miR-125a plays an important role in carcinogenesis, however, the role of miR-125a in hepatocellular carcinoma (HCC) remains elusive.

**Methodology/Principal:**

Real time-PCR (qRT-PCR) was performed to test the significance of miR-125a in HCC. Ectopic expression of miR-125a was used to test the influences of miR-125a on proliferation and metastasis of HCC cells *in vitro* and *in vivo*. Predicted target genes of miR-125a were determined by dual-luciferase reporting, qRT-PCR, and western blot (WB) analyses. Then immunohistochemical staining (IHC) was used to detect the expression of target genes, and the correlations and prognostic values of miR-125a and its target genes were also investigated.

**Conclusions/Significance:**

Decreased miR-125a was observed in both HCC tissues and cell lines, and associated with patients’ aggressive pathologic features. Up-regulating miR-125a significantly inhibited the malignant phenotypes by repressing the expression of matrix metalloproteinase 11 (MMP11) and vascular endothelial growth factor A (VEGF-A) both *in vitro* and *in vivo*. Furthermore, miR-125a expression was inversely correlated with both MMP11 and VEGF-A expression in HCC tissues. Inhibiting miR-125a could increase both MMP11 and VEGF-A expression, and RNA interference targeting MMP11 or VEGF-A mRNA could rescue the loss of miR-125a functions. MiR-125a inhibits the proliferation and metastasis of HCC by targeting MMP11 and VEGF-A. Up-regulation of miR-125a might be a promising approach and a prognostic marker for HCC.

## Introduction

Hepatocellular carcinoma (HCC) is the second most frequent cause of cancer death worldwide, and half of HCC diagnoses and deaths were estimated to occur in China [1]. Despite improvements in surgery and other treatments, the postoperative prognosis of HCC patients is still unsatisfactory due to the high rate of recurrence and metastasis. Therefore, identifying the malignant factors and understanding the mechanisms are critical for the improvement of therapeutic strategies for HCC patients.

MicroRNAs (miRNAs) are small non-protein-coding RNAs of 20–24 nucleotides that can down-regulate the expression of their target genes through binding to the 3′ untranslated regions (UTRs) of target mRNAs, resulting in mRNA degradation and translation inhibition [2], [3]. Several miRNAs have been identified to be abnormally expressed, and to be associated with the malignant progressions of various human cancers [4], [5], [6]. MiRNAs were recently found to be frequently down-regulated in HCC, and they were also shown to be associated with the carcinogenesis, metastasis, recurrence and prognosis of HCC [7], [8], [9], [10]. However, further investigation is needed to define the molecular mechanisms underlying the effects of miRNAs on the development of HCC. Previous studies have demonstrated that several miRNAs that are located in fragile sites or aberrant genomic regions play oncogenic or suppressive roles in the progression of human cancers [4]. MiR-125a is located at 19q13, which is frequently deleted in several human cancers. MiR-125a over-expression impaired the anchorage-dependent growth, migration and invasion of breast cancer cells by down-regulating ERBB2 and ERBB3 in the ERBB2-dependent SKBR3 cell line [11]. Researchers showed that miR-125a could inhibit the proliferation of human gastric cancer cells in combination with trastuzumab [12]. Furthermore, miR-125a was also down-regulated in non-small cell lung cancer and had inverse effects on the invasion and migration of lung cancer cells [13]. Overexpression of miR-125a induces conversion of highly invasive ovarian cancer cells from a mesenchymal to an epithelial morphology, suggesting that miR-125a is a negative regulator of EMT [14]. However, the expression and roles of miR-125a in HCC have not been studied, nor the prognostic value and co-expression of miR-125a with target genes in tumor samples from patients have rarely been investigated.

In the present study, we found that the miR-125a was frequently down-regulated in HCC compared with matched adjacent liver tissues, and was correlated with the malignant progressions of patients. Ectopic expression of miR-125a could repress the proliferation and metastasis of HCC cell lines both *in vitro* and *in vivo*. However, over-expression of miR-125a could not significantly down-regulate the expression of ERBB2 or ERBB3 in HCC cells. Therefore, further studied were needed to elucidate the mechanisms underlying the inhibitory effects of miR-125a on HCC cells, and the prognostic values and co-expression of miR-125a with target genes in tumor samples from patients were also investigated.

**Table 1:** Primers for 3′UTR of target genes.

Target genes	Primers
MMP11	F 5′CTAG ACTAGT CGAGCCTGCCAACACT 3′
	R 5'CCC AAGCTT GGGATACAGCAAGGACAC 3′
ERBB2	F 5'CTAG ACTAGT AGGGAAGAATGGGGTCGT 3′
	R 5′ CCC AAGCTT GATGCCAGCAGAAGTCAGG 3′
EDN1	F 5' CTAG ACTAGT TGGGGATGACAATGGACC 3'
	R 5' CCC AAGCTT TGGGAAGAGTTTGGGGAG 3'
VEGF	F 5′ CTAG ACTAGT AGATGTGACAAGCCGAGG 3′
	R 5′ CCC AAGCTT GTCTACAGGAATCCCAGAAATA 3′
ERBB3	F 5̀CTAG ACTAGT GGCAACGAGATGGAGGTGGT 3′
	R 5′ CCC AAGCTT GGGAATAGGGAGAAGACGGT 3′
MMP14	F 5′ CTAG ACTAGT TGCTCTACTGCCAGCGTTCC 3′
	R 5' CCC AAGCTT TGACCCCATTTGACCTTTA 3'
BCL9L	F 5' CTAG ACTAGT AATCGGGGAAACAGCATC 3'
	R 5' CCC AAGCTT GGACAAACACCCAGTCGTAT 3'
VEGF-Del	F 5' GAAGGAGCCTCCTTTCGGGAACCAGATCT 3'
	R 5' TGGTTCCCGAAAGGAGGCTCCTTCCTCCT 3'
MMP11-Del	F 5' ATATTCGTGGCCTGGCAGGGGTCAGCA 3'
	R 5' GTCAGCATCAGAGGAAAGTGTTGGCA 3'

## Materials and Methods

### Cell Lines and Human Samples

Human HCC cells (SMMC7721, MHCC97L, MHCC97H and HCCLM3) were kindly provided by Dr. Tang ZY (Liver Cancer Institute, Zhongshan Hospital, Fudan University, Shanghai, China) [15]. HepG2 cell line was obtained from American Type Culture Collection (ATCC number: HB-8065, Manassas, VA). QZG cell line was obtained from the Cell Research Institute of the Chinese Academy of Sciences (Shanghai, China**)**, and both of them are maintained in our lab [16]. All of the cell lines were cultured in Dulbecco’s modified Eagle medium (DMEM) supplemented with 10% FBS, 100 µg/ml penicillin, and 100 µg/ml streptomycin at 37°C in a 5% CO_2_ incubator. Fresh cancerous and matched adjacent liver tissues were obtained from 80 patients who received primary HCC resection between January 2006 and December 2006 at the Department of General Surgery in Xijing Hospital (Xi’an, China). The tissues were immediately frozen in liquid nitrogen after surgical removal and stored at −80°C until use. Paraffin-embedded HCC tissues from the 80 patients were also obtained from Department of Pathology at Xijing Hospital. This study was approved by the Hospital’s Protection of Human Subjects Committee, and informed consent was also obtained from all patients.

**Table 2:** Primers for qRT-PCR.

Genes	Primers
MMP11	F 5′ TCCTGACTTCTTTGGCTGTG 3′
	R 5′ CCATGGGTCTCTAGCCTGAT 3′
MMP9	F 5′ TCCAGTACCGAGAGAAAGCC 3′
	R 5′ GCAGGATGTCATAGGTCACG 3′
MMP14	F 5' CACAAGGACTTTGCCTCTGA 3'
	R 5' CAGAGAGAAGCAAGGAGGCT3'
VEGF	F 5′ TCCCGGTATAAGTCCTGGAG 3′
	R 5′ ACAAATGCTTTCTCCGCTCT 3′
BCL9L	F 5'GCTTCCCTCTCTCTTTCCCT 3′
	R 5' CCTCTCAGAGCCAAGAGGAC 3'
MMP2	F 5' TGAGAAGGATGGCAAGTACG3'
	R 5' TCAGTGGTGCAGCTGTCATA3'
ERBB2	F5′ CAGAGTACCATGCAGATGGG 3′
	R 5′ CATCACTCTGGTGGGTGAAC 3′
GAPDH	F5′ GCACCGTCAAGGCTGAGAAC 3′
	R 5′ TGGTGAAGACGCCAGTGGA 3′

### RNA Extraction and Real Time-PCR Analysis (qRT-PCR)

Total RNA was extracted from cell lines and tissue samples using Trizol (Invitrogen), and the concentration of total RNA was quantitated by measuring the absorbance at 260 nm. The miScript SYBR^®^ Green PCR Kit from QIAGEN was used for qRT-PCR analysis. Primers of miR-125a and U6 snRNA (internal control) were also purchased from QIAGEN (MS00003423 and MS00033740). The fold-change for mRNA in HCC tissues relative to the adjacent non-tumor (NT) tissues was calculated using the 2^−ΔΔCT^ method [17], where ΔΔCt = ΔCt HCC/NT and ΔCt = Ct miR-125a-Ct U6. The relative mRNA levels of genes in HCC cells were also determined by qRT-PCR, and the expression of GAPDH was used as the internal controls. Each PCR was performed in triplicate. The primers for the genes examined were presented in Table 1.

### Plasmids Construction and Transfection

Lentivirus-mediated miR-125 was constructed as described in a previous study [17]. The precursor sequence of miR-125a was generated as follows: (Forward) 5′-CTATGTTTGAATGA GGCTTCAG-3′ and (Reverse) 5′-CGCGTCGCCGCGTGTTTAAACG-3′ [18]. The sequence targeting MMP11 for mRNA interference was 5′-ATGTCCACTTCGACTATGATG-3′; targeting VEGF-A was (5′-AUGUGAAUGCAGACCAAAGAA-3′), and targeting mature miR-125a was (5′-UCCCUGAGACCCUUUAACCUGA-3′ ). These siRNAs were synthesized and inserted into the Pgene sil-1 vector. The empty vector (without miR-125a or siRNA) was used as a negative control. All of the vectors also encoded green fluorescent protein (GFP).

The miR-125a or the negative control virus was infected into HepG2 and HCC-LM3 cell lines respectively. The siRNA targeting the mature miR-125a and control were transfected into SMMC7721 cells, which showed relatively high miR-125a expression in HCC cell lines, and the successfully transfected cells were named si-7721 and con-7721. The successfully transfected, GFP-positive cells were separated by flow cytometry (FACScan; Becton Dickinson, San Jose, CA). The purified, GFP-positive cell lines were named HepG2-125a, HepG2-NC, LM3-125a, LM3-NC, Si-7721 and Con-7721. Then MMP11, VEGF-A and the control siRNA were transfected into si-7721 cells, and named Si-MMP11, Con-MMP11, Si-VEGF and Con-VEGF.

### MTT Assay

MTT assays were performed to evaluate the speed of cell proliferation as described previously [19]. Briefly, log phase cells were trypsinized into a single cell suspension and seeded into 96-well plates at a density of 1×10^3^ cells/well. After 1, 2, 3, 4, 5, 6 or 7 days of cell cultivation, 20 µl of 3-(4,5)-dimethylthiahiazol (-z-y1)-3,5-di-phenytetrazo-libromide (MTT, 5 mg/ml; Sigma, St. Louis, MO) was added into each well and incubated for 4 hours at 37°C. The supernatant was then aspirated, and 150 µl dimethylsulfoxide (DMSO) was added to dissolve the crystals with agitation for 10 minutes at room temperature. The absorbance values were measured using an ELISA reader (Bio-Rad Laboratories, Richmond, CA) at a wave length of 490 nm. Each experiment was repeated three times.

**Figure 1:**
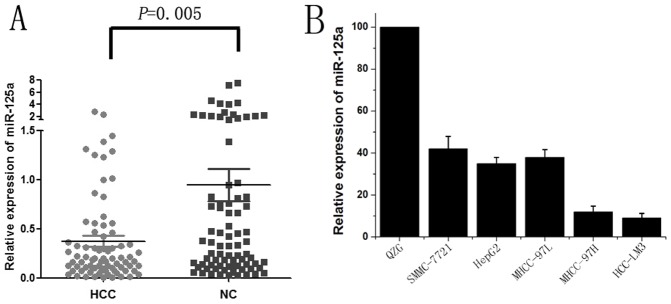
Down-regulation of miR-125a in HCC tissues and cell lines. The expression of miR-125a in tissues and cell lines was detected by qRT-PCR analysis, and the results shown are the mean value of three repetitions calculated by 2^−△△Ct^ with U6 snRNA as the internal control. *(A)* MiR-125a expression in 80 HCC and paired adjacent non-tumor liver tissues. A significantly lower expression level of miR-125a was observed in HCCs compared with that in adjacent liver tissues (*P* = 0.005). *(B)* Relative fold changes of miR-125a in HCC cell lines compared to the normal Liver cell line QZG.

**Table 3:** MiR-125a and MMP11 expression correlated with clinical features of patients.

		MiR-125a		*P* value	MMP11		*P* value
Parameter	Group	Low	High		–	+	
Age	<56	20	20	1.00	18	22	0.36
	>56	20	20		14	26	
Gender	Male	28	28	0.64	18	36	0.08
	Female	12	14		14	12	
HBV	−	5	9	0.24	9	5	0.05
	+	35	31		23	43	
Metastasis	Without	17	32	0.001	26	23	0.003
	With	23	8		6	25	
AFP	Low	19	21	0.37	18	22	0.36
	High	21	19		14	26	
T stage	I+II	10	27	<0.001	19	18	0.06
	III+IV	30	13		13	30	

The associations of miR-125a and MMP11 with clinical features were detected by Kruskal-Wallis *H* or Mann–Whitney *U* test. *P < 0.05 was considered statistically significant.

### Soft Agar Assay

Soft agar assays were performed to evaluate the in vitro tumorigenicity of cells. Approximately 2×10^3^ cells from each cell line were seeded in 0.3% agarose with a 0.5% agarose underlay in a six-well plate. The cells in agarose were cultured at 37°C in an atmosphere of 5% CO_2_ at mosphere for 17 days. Cell colonies were counted by two independent observers. Each cell line was tested in triplicate, and the results shown are the mean of three repeated experiments.

**Figure 2:**
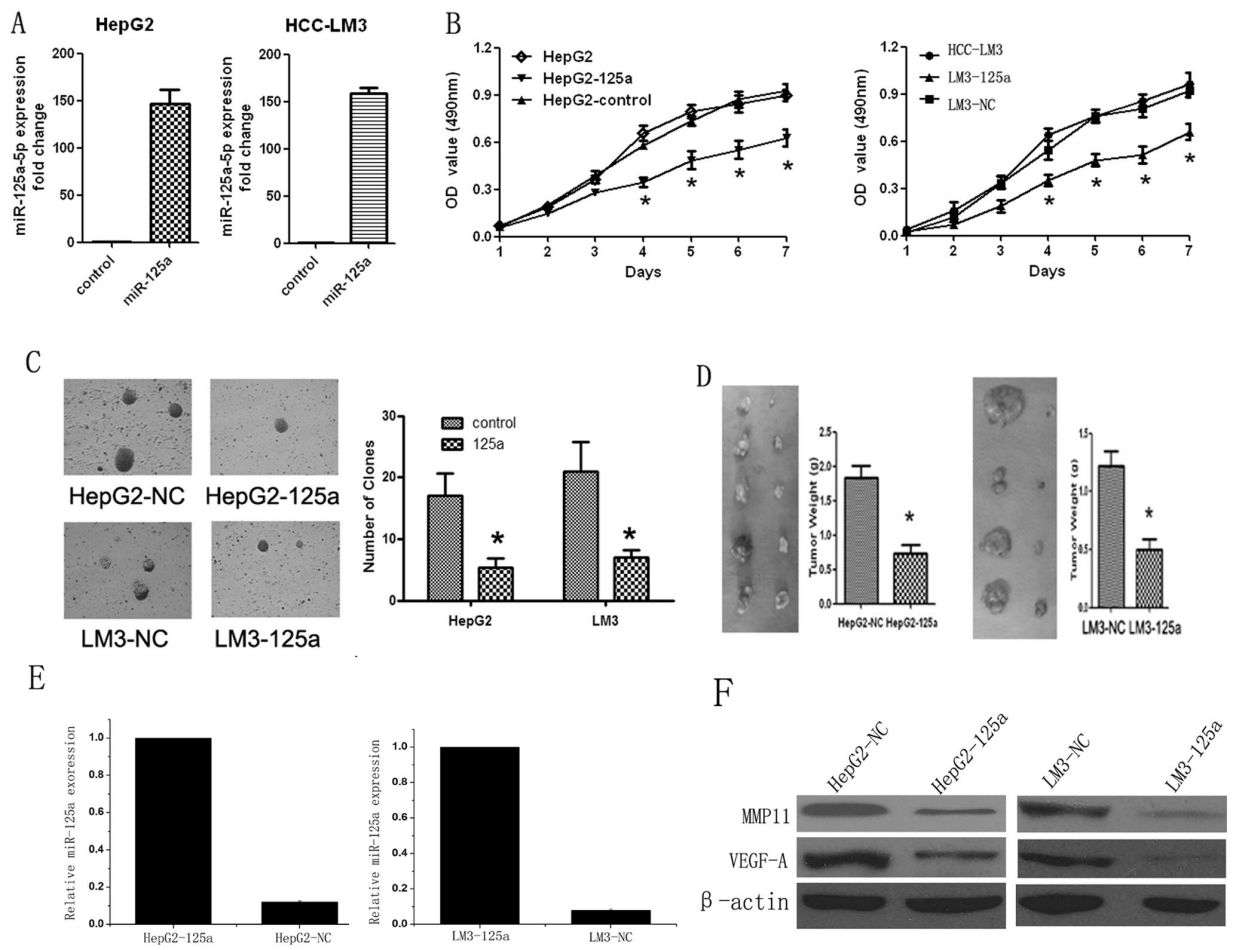
Inhibition of cell proliferation by ectopic expression of miR-125a *in vitro* and *in vivo*. (A) qRT-PCR analysis confirmed that miR-125a expression was significantly up-regulated in HepG2-125a and LM3-125a cells compared with matched controls. U6 snRNA was used as the internal control. (B) The growth curves are plotted based on the MTT assay results. The data presented are the mean value of three repetitive determinations. (C) The colony numbers shown are the mean of three repetitions, and are expressed as the mean ± SD. (D) In vivo tumorigenicity in nude mice. HepG2-125a or LM3-125a cells were injected into the right flanks and their matched controls were injected into the left flanks of mice. Four weeks later, the tumors were resected from the mice, and calculated using the formula: L×S2 /2. (E and F) qRT-PCR and western blot showed that the expression of miR-125a in the tumor from right flank were significantly lower that the left flank. Inversely, both the expression of MMP11 and VEGF-A were significantly higher in the right flank were significantly lower that the left flank (Fig. 2 E and F). *P<0.05 *_VS_* matched controls.

### Cell Invasion and Migration Assays

Cell migration and invasion assays were performed as described by others [20]. For migration assays, HepG2-125a, HepG2-NC, LM3-125a and LM3-NC cells were cultured in DMEM with 0.1% FBS. After 24 h, 2×10^5^ starved cells in DMEM without FBS or HGF were plated in the upper chamber with a non-coated membrane (6.5 mm in diameter, 8-lm pore size; Corning, NY, USA). For invasion assays, the chambers used were coated with 200 mg/ml of Matrigel and dried for 12 h. Then, 2×10^5^ cells were added to the top chamber. For both invasion and migration assays, medium with 10% FBS and HGF (20 ng/ml) was placed into the lower chamber. After 24 h at 37°C, the cells on the upper membrane surface were carefully removed. After incubation with 95% ethanol for 20 min, the filters were stained with 0.2% crystal violet solution for 10min, and counted using an inverted microscope. The results presented are the mean of three independent assays.

**Figure 3:**
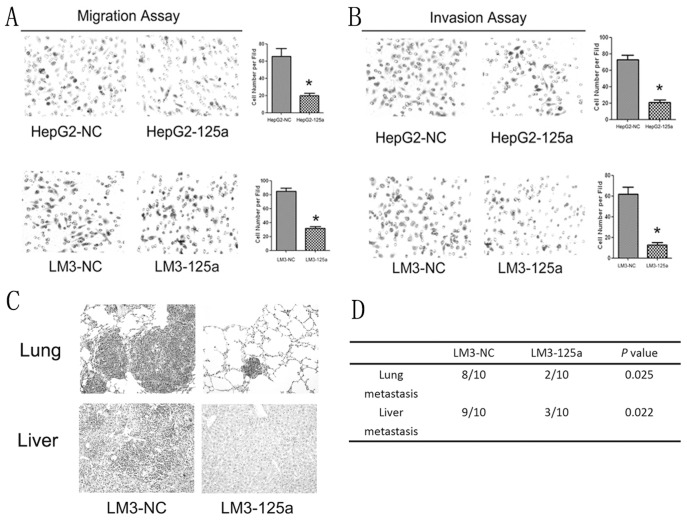
Suppression of cell invasion and metastasis *in vitro* and *in vivo* by over-expression of miR-125a. (A and B) Migration and invasion assays confirmed that the invasive abilities of HepG2 and HCC-LM3 cells were significantly inhibited by ectopic expression of miR-125a. The data presented are the mean of three independent experiments. *P<0.05 *_VS_* each control. (C) LM-125a and LM3-NC cells were used for the *in vivo* metastasis assays. The pictures shown are the representative anatomical photos of livers and lungs from mice injected with LM-125a or LM3-NC cells. (D) The data presented are the incidences of metastasis in both the livers and lungs of mice.

**Figure 4:**
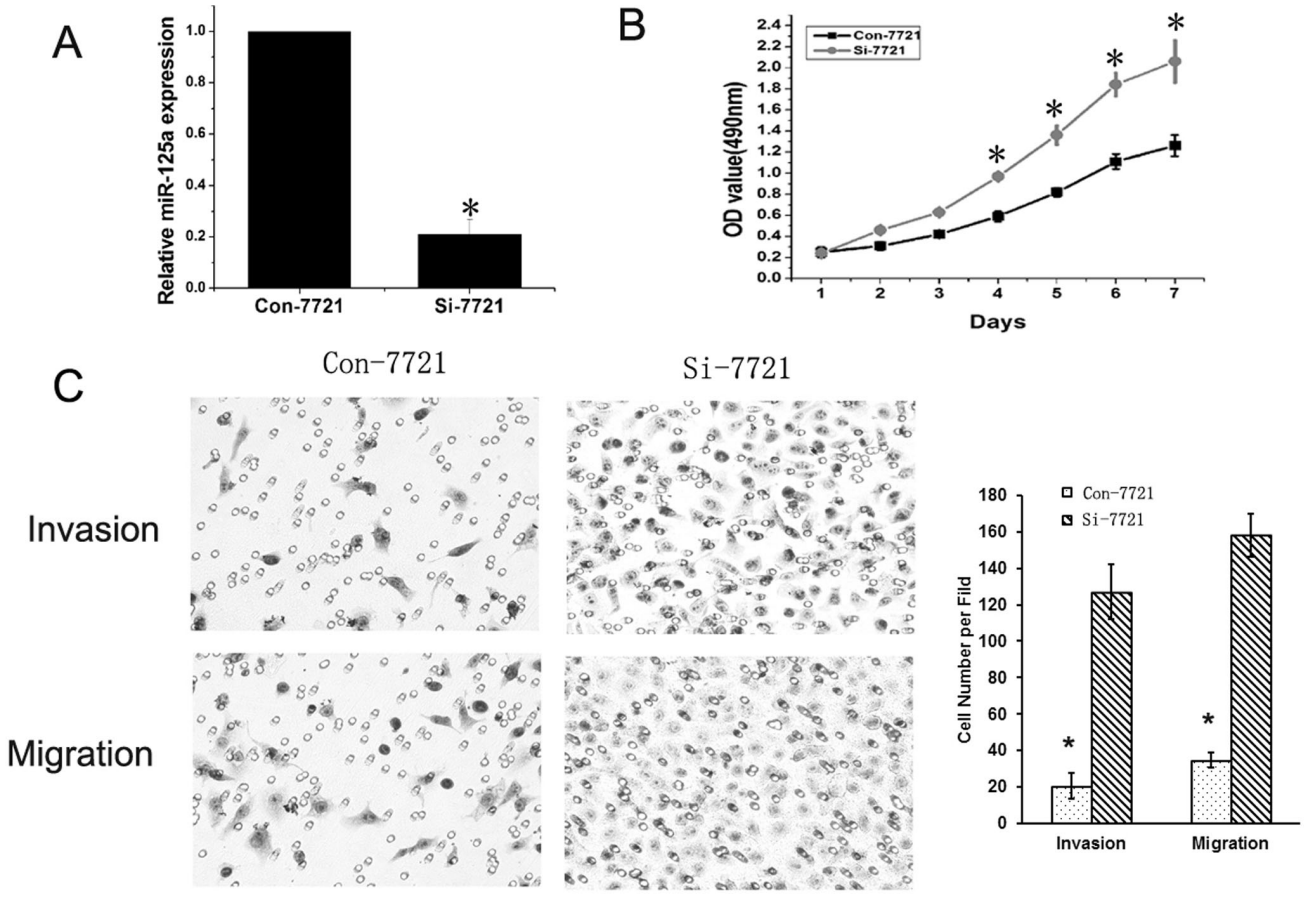
Inhibition of miR-125a promoted the proliferation and invasion of SMMC-7721 cells. (A) qRT-PCR analysis showed that miR-125a was significantly down-regulated by lentivirus mediated siRNA targeting mature miR-125a. (B) Down-regulation of miR-125a could significantly increase the growth of SMMC-7721 cells. (C) Both invasion and migration assays demonstrated that down-regulation of miR-125a could significantly promote the migration and invasion of cells.

### Luciferase Reporter Assay

For dual luciferase assays, HepG2-125a or HepG2-NC cells in 24-well plates were co-transfected with 0.4 µg of the firefly luciferase report vector and 0.08 µg of pRL-TK control vector containing Renilla luciferase (Promega) using siPORTneoFX (Ambion, Austin, TX) according to the manufacturer's protocol. Lysates were prepared at 48 h post-transfection. Luciferase activity was measured using the Dual-Light luminescent reporter gene assay (Applied Biosystems). Firefly luciferase activity was normalized to renilla luciferase activity for each transfected well. Three independent experiments were performed in triplicate. The primers for the 3′-UTR of targets containing a miR-125a binding site are shown in Table 2.

### Western Blot Analysis

Western blot assays were performed as described in our previous study [19]. Total protein was separated using sodium dodecyl sulfate–polyacrylamide gel electrophoresis and transferred to nitrocellulose membrane (Bio-Rad, Hercules, CA). The membranes were blocked with 10% nonfat milk in TBS-T and incubated with mouse anti-MMP11 (Cell Signaling Technology, 1∶300), rabbit anti-VEGF (Santa Cruz, 1∶200), rabbit anti-ERBB2 (Cell Signaling Technology, 1∶500), rabbit anti-ERBB3 (Cell Signaling Technology, 1∶500) or mouse anti-β-actin (Sigma,1∶4000) antibodies. The levels of goal protein were detected with enhanced chemiluminescence reagents (Pierce, Rockford, IL). β-actin was used as an internal control.

### Immunohistochemical Staining (IHC)

IHC was used to detect the MMP11 and VEGF expression in HCC tissues as described in our previous work [19]. After being blocked with 3% H_2_O_2_, sections were incubated overnight at 4°C with antibodies (MMP11, 1∶50;VEGF, 1∶100) diluted in phosphate buffered saline (PBS). After three 5-min washes with PBS-T, sections were incubated with biotinylated goat anti-Rb or mouse IgG/HRP (1∶2000, Zhongshan) for 1h, followed by three additional 5-min washes with PBS-T. DAB solution was used for visualization of the samples. As a negative control, the primary antibody was replaced with PBS. As in previous study, scores of 0 and 1 were considered to represent negative expression, and 2 and 3 were considered to represent positive expression [11].

**Figure 5:**
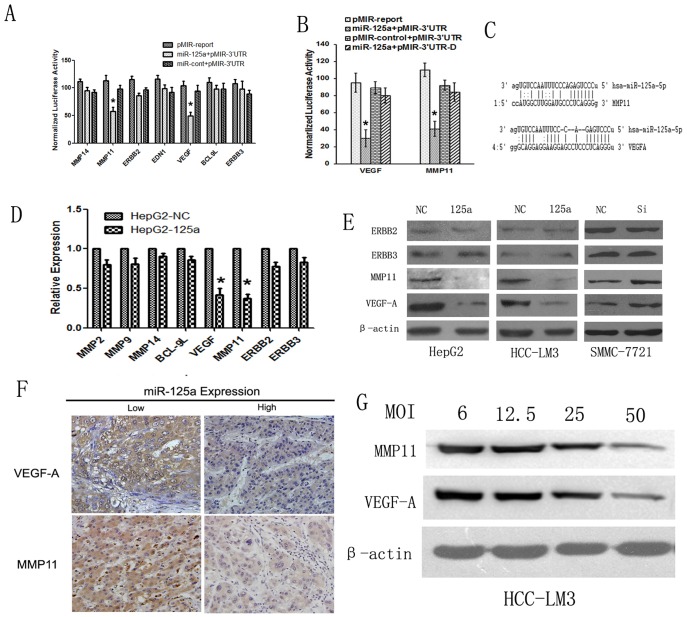
MiR-125a inhibits cell proliferation and metastasis by directly targeting VEGF-A and MMP11. (A) Dual luciferase assays were performed in HepG2-125a and HepG2-NC cells transfected with the firefly luciferase report and the control vectors containing Renilla luciferase. The result showed that miR-125a could significantly suppress the luciferase activity of reporters containing the 3′UTRs of MMP11 and VEGF**,** but has no significantly effect on reporters containing the 3′UTRs of Bcl-9L, EDN1, MMP14, Her2, ERBB2 or ERBB3 in HCC cells. The data shown are the means±SE of three independent experiments. (B) Assessment of the specificity of binding site. A Reduction of luciferase activity driven by the MMP11 and VEGF 3′UTR constructs was observed in HepG2-125a cells co-transfected with pMir-3′UTR, but not in cells co-transfected with pMIR-3′UTR-D. (C) The sequences of the miR-125a binding sites within the VEGF-A and MMP11 3′UTRs were presented. (D) qRT-PCR analysis shows that the VEGF-A and MMP11 mRNAs, but not others, were significantly decreased in HepG2-125a cells compared with HepG2-NC cells. (E) Western blot analysis confirmed that miR-125a significantly inhibited the endogenous expression of VEGF-A and MMP11, but not ERBB3 or ERBB2. β-actin was used a the loading control. (F) Down-regulation of miR-125a was significantly associated with high expression levels of VEGF-A and MMP11 in HCC clinical samples. Positive VEGF-A and MMP11 expression was observed in the samples with low miR-125a expression (left), and negative staining for VEGF-A and MMP11 was observed in samples with high miR-125a expression (right). (G) Western blot analysis shows that miR-125a exhibited a dose-dependency on MMP11 and VEGF-A expression in HCC-LM3 cells, and the MOI of 50 showed the best effect. *P<0.05 *_VS_* control.

### Animal Experiments

Athymic mice (4–5 weeks old) were purchased from the Animal Center of the Chinese Academy of Science (Shanghai, China) and maintained in laminar ﬂow cabinets under specific pathogen-free conditions. A total of 10 mice were used for the subcutaneous study. HepG2-125a or LM3-125a cells were injected into the right ﬂanks, and HepG2-NC or LM3-NC cells were injected into the left flanks. Four weeks after inoculation, the tumor-bearing mice were sacrificed, and the tumor volumes were calculated following the formula: L×S2 /2. Moreover, the total RNA and protein of each representative tumor from mice were used for analyses of the expression levels of miR-125a, MMP11 and VEGF-A target genes by qRT-PCR and western blot.

Previous studies have demonstrated that HCC-LM3 is a highly invasive cell line that can be used for *in vivo* metastasis assays [15], therefore, LM3-125a and LM3-NC cells were chosen for the metastasis experiments. Approximately 2×10^6^ cells from each cell line were suspended in 0.2 ml sterile PBS and injected into the tail veins of 10 mice. The mice were sacrificed 6 weeks after injection. After the visible tumors numbers in the liver and lung surface were counted, histological examinations were performed.

### Statistical Analysis

All statistical analyses were carried out using the SPSS 16.0 software package (SPSS, Chicago, IL), and variables with a value of *P*<0.05 were considered to be statistically significant. The differential expression of miR-125a in HCC and paired noncancerous tissues was analyzed using the Wilcoxon rank sum test. The Kruskal–Wallis *H* test or the Mann–Whitney *U* test was used to investigate the significance of miR-125a, VEGF-A and MMP11 expression as correlated with clinical features in HCC. A one-way ANOVA test was utilized to evaluate the difference between three comparisons in cell proliferation and soft agar clonogenic assays, and the least significant difference-T test was used for the analysis of two groups. Overall survival curves were plotted using the Kaplan-Meier method and were evaluated for the statistical significance using a log-rank test.

**Table 4:** The correlations of miR-125a with MMP11 and VEGF-A expression in HCC tissues.

		MMP11 expression		R value	P value	VEGF expression		R Value	P value
		−	+			−	+		
miR-125a	Low	8	32	−0.41	<0.01	2	38	−0.59	<0.01
expression	High	24	16			24	16		

R: regression coefficient as evaluated by Pearson Correlation. *P* < 0.05 was considered statistically significant.

**Figure 6:**
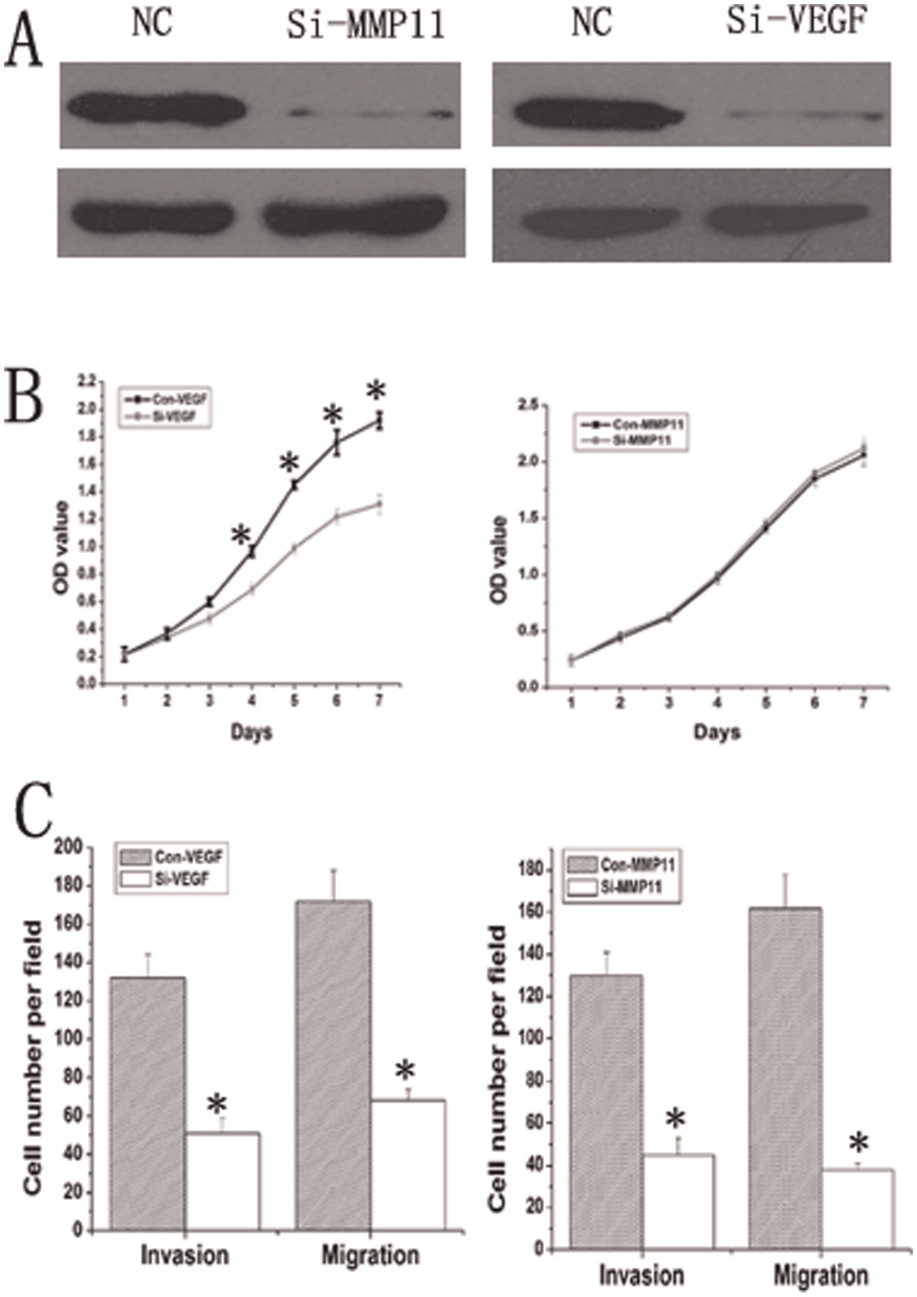
Down-regulation of MMP11 or VEGF-A rescued the loss of miR-125a function in Si-7721 cells. (A) Western blot showed that exprssion of MMP11 or VEGF-A was significantly repressed by each siRNA transfection. (B) MTT assays showed that knockdown VEGF could significantly inhibit the growth of Si-7721 cells, but inhibition of MMP11 could not significantly influence the growth of Si-7721 cells. (C) RNA interference targeting MMP11 or VEGF-A mRNA could significantly repress the migration and invasion of Si-7721 cells.

## Results

### MiR-125a was Down-regulated in HCC Tissues and Cell Lines

The expression of miR-125a in HCC and paired adjacent non-tumor liver tissues from 80 patients was detected using qRT-PCR. The results showed that miR-125a expression was decreased in 77.5%(62/80)of HCC tissues compared with matched adjacent liver tissues, with an average of 4.72-fold reduction in expression (median =  0.37 vs 0.95; *P*  =  0.005; Fig. 1A). Further analysis revealed that lower expression of miR-125a in HCC was significantly correlated with the patients’ clinical features, including T stage and tumor metastasis (Table 3; *P* < 0.05).

Consistent with the results observed in tissues, significantly decreased expression of miR-125a was observed in all five HCC cell lines (SMMC-7721, HepG2, MHCC-97L, MHCC-97H and HCC-LM3) compared with the QZG cells (Fig. 1B). MiR-125a expression was low in SMMC-7721 and HepG2 cells, but barely in highly invasive cell lines, such as MHCC-97L, MHCC-97H and HCC-LM3. Therefore, we chose the HepG2 and HCC-LM3 cell lines for further study.

**Figure 7:**
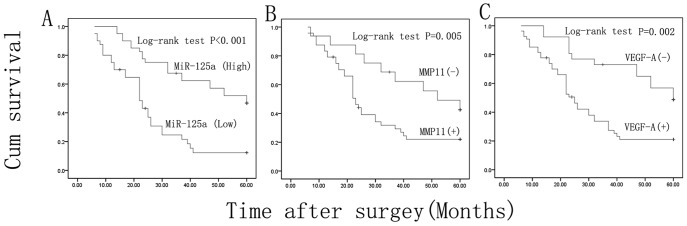
Kaplan-Meier survival analysis and log-rank test for HCC patients. (A) Lower miR-125a expression, (B) higher MMP11 or (C) VEGF-A, is correlated with poor 5-year cumulative survivals of the 80 HCC patients.

**Table 5:** Univariate and multivariate analyses of clinical parameters associated with prognosis.

Parameter	Chi-square	P value
Age	0.38	0.54
Gender	0.03	0.86
HBV	0.41	0.52
Metastasis	14.6	<0.001
AFP	9.4	0.002
T stage	5.38	0.02
miR-125a	17.9	<0.001
MMP11	7.85	0.005
VEGF-A	9.91	0.002

The univariate analysis shows that metastasis, AFP, T stage, miR-125a, MMP11 and VEGF-A are statistically significant prognostic factors for HCC patients.

### Ectopic Expression of MiR-125a Inhibited HCC Cell Proliferation

To investigate the effects of miR-125a on the proliferation of HCC, HepG2 and HCC-LM3 cell lines were used for the lentivirus-mediated miR-125a transfection. As presented in Figure 2A, qRT-PCR analysis confirmed that miR-125a was markedly up-regulated in both HepG2-125a and LM3-125a cells compared with controls. MTT assays were performed to test the effects of miR-125a on cell growth. Both HepG2-125a and LM3-125a cells exhibited much slower growth than their corresponding controls, and the inhibitory effects showed statistical significance after culture for four days (Fig. 2B). The colony formation assay results showed that ectopic expression of miR-125a markedly decreased the colony numbers of HepG2 and HCC-LM3 cells (Fig. 2C; *P*<0.01). These data were further strengthened by the results from the nude mice tumorigenicity experiments. Four weeks after subcutaneous injection, tumors appeared much smaller at the sites injected with HepG2-125a or LM3-125a cells compared with each control (Fig. 2D; *P* < 0.01). Thus, the over-expression of miR-125 may inhibit the tumorigenicity of HCC cells *in vitro* and *in vivo*. Moreover, the total RNA and protein of each representative tumor from mice were used for analyses of the expression levels of miR-125a, MMP11 and VEGF-A target genes by qRT-PCR and western blot. The result showed that the expression of miR-125a in the right flanks were significantly lower that the left flanks. Inversely, both the expression of MMP11 and VEGF-A were significantly higher in the right flanks were significantly lower that the left flanks (Fig. 2 E and F).

### Over-expression of MiR-125a Repressed HCC Cell Invasion and Metastasis

Because the low expression levels of miR-125a in HCC patients were markedly correlated with HCC metastasis, we postulated that increased expression of miR-125a could inhibit the invasion and metastasis of HCC cells. As shown in Figures 3A and 3B, over-expression of miR-125a significantly inhibited the migratory and invasive abilities of HepG2 and HCC-LM3 cells (*P* < 0.01). Consistent with the *in vitro* observations, the animal experiment results showed that liver and lung metastases were apparent in mice injected with LM3-NC cells, but few were observed in mice injected with LM3-125a cells (Figure 3C). Histological analysis revealed that both the number and the size of metastatic nodules in the lungs and livers of mice were significantly lower than those in the controls (Figure 3D; *P* < 0.01). Therefore, miR-125a could repress the metastasis of HCC both *in vitro* and *in vivo*.

### Down-regulation of miR-125a Inceased the Proliferation and Invasion of SMMC-7721 Cells

To study the effects of miR-125a down-regulation on proliferation and invasion of HCC, lentivirus-mediated siRNA targeting the precursor of miR-125a was transfected into SMMC-7721 cells, which showed relative high miR-125a expression among HCC cell lines (Figure 1B). qRT-PCR analysis showed that miR-125a was significantly deceased in Si-7721 cells (Figure 4A). The results of MTT revealed that miR-125a inhibition could promote the growth of SMMC-7721 (Figure 4B). Both migration and invasion assays showed that the migratory and invasive abilities of Si-7721 cells were significantly lower compared with controls (Figure 4C).

### MiR-125a Directly Down-regulated the MMP11 and VEGF-A

To explore the mechanism underlying miR-125a involvment in the progression and metastasis of HCC, miRanda and Pic Tar algorithms were used to search for the target genes. The analysis revealed several tumorigenicity-related genes as potential targets of miR-125a, including MMP11, ERBB2, ERBB3,EDN1, VEGF-A, MMP14 and BCL-9L. Therefore, we constructed luciferase reporters carrying the 3′-UTR with the putative miR-125a binding sites for each of those genes. Luciferase assays showed that the 3′-UTRs of both MMP11 and VEGF-A, but not others, caused a significant reduction in the luciferase activity (Figure 5A); however, these effects disappeared with the deletion of key seed regions in the 3′UTR of MMP11 or VEGF-A (Figure 5B). The miR-125a binding sites in the 3′UTRs of MMP11 and VEGF-A are shown in Figure 5C. The qRT-PCR analysis showed that the mRNA expression levels of MMP11 and VEGF-A, were significantly inhibited by the over-expression of miR-125a (Figure 5D). These findings were further verified by the results of western blot analyses, which revealed that over-expression of miR-125a could markedly suppress MMP11 and VEGF, but not ERBB2 or ERBB3, expression in HepG2 and HCC-LM3 cells, and down-regulation of miR-125a could obviously increase the expression of MMP11 and VEGF in SMMC-7721 cells (Figure 5E). Then HCC-LM3 cells were infected with the miR-125a lentivirus in different MOI. Western blot analysis showed that miR-125a exhibited a dose-dependency on MMP11 and VEGF-A expression in HCC-LM3 cells (Figure 5G).

We also asked whether miR-125a was correlated with MMP11 and VEGF-A expression in clinical HCC tissues. IHC was performed to detect MMP11 and VEGF-A expression in HCC samples from the 80 patients. Spearman’s rank test showed that a significant negative correlation was found between miR-125a and MMP11 protein expression (r = −0.41, *P* < 0.01),and down-regulation of miR-125a was also strongly correlated with up-regulation of VEGF-A expression in these HCC tissues (r = −0.59, *P* < 0.01) (Figure 5F; Table 4).

### Down-regulation of MMP11 or VEGF-A Rescued the Loss of miR-125a Function

The expression of MMP11 and VEGF-A were markedly up-regulated in si-7721 cells comapared with SMCC7721 cells (Figure 5E ). To investigate whether miR-125a works through MMP11 or VEGF-A, the siRNA of MMP11 or VEGF-A was transfected into Si-7721 cells. Western blot showed that MMP11 or VEGF-A was significantly down-regulated in the Si-MMP11 or Si-VEGF cells compared with Si-7721 cells (Figure 6A). MTT assays showed that knockdown VEGF could significantly inhibit the growth of Si-7721 cells, but inhibition of MMP11 could not significantly influence the growth of Si-7721 cells (Figure 6B). What’s more, RNA interference targeting MMP11 or VEGF-A mRNA could significantly repress the migration and invasion of Si-7721 cells (Figure 6C). These data indicated that MMP11 or VEGF gene silencing could rescue the loss of miR-125a function in SMMC-7721 cells.

### Survival Analysis

MiR-125a, MMP11 and VEGF-A expression levels in HCC samples were used for a survival analysis. The median survival time of these patients was 32 months after operation. A Kaplan-Meier analysis revealed that low miR-125a expression was significantly associated with poor 5-year overall survival time (25.7±2.7 months *vs* 45.4±2.8 months, *P* < 0.001) (Fig. 7A), and that higher MMP11 expression was another significant prognostic factor for poor survival (44.7±3.3 months *vs* 29.7±2.7 months, *P*  = 0.005) (Fig. 7B). Interestingly, higher expression of VEGF-A was also correlated with poor survival of these patients (29.8±2.5 months *vs* 48±3.4 months, *P* = 0.002) (Fig. 7C). Univariate analysis showed that the patients’ survival time was also significantly correlated with the clinical features such as metastasis (*P* < 0.001), AFP (*P* = 0.002) and T stage (*P* = 0.02), but not with age, gender or HBV infection (Table 5; *P* > 0.05). Multivariate regression analysis showed that AFP, but not others, was an independent prognostic factor.

## Discussion

Several studies have revealed that the expression of miRNAs is frequently down-regulated and correlated with the malignant progression of HCC. However, because they can post-transcriptionally regulate hundreds of targets involved in several signal transduction pathways, the roles of miRNAs in the carcinogenesis of HCC are far from being fully be elucidated. Therefore, exploring the clinical potential and molecular mechanism of miRNAs underlying malignant development is significant for beneficial therapies to delay the development and ameliorate the living conditions of HCC patients.

Previous studies have shown that miR-125a expression was down-regulated in several different tumors, including breast cancer, ovarian cancer, lung cancer, medulloblastoma and gastric cancer [11], [13], [14], [21]. The results of human miRNA microarrays have indicated that HCC tissues exhibit lower expression of miR-125a [22], [23]. These studies rarely focused on the prognostic values and co-expression of miR-125a with target genes in tumor samples from patients. In the present study, we demonstrated for the first time that miR-125a was frequently down-regulated in HCC compared with matched adjacent liver tissues, and that lower expression of miR-125a was significantly associated with the progression and poor prognosis of patients with HCC. Consistent with the results observed in tissues, significantly lower expression of miR-125a was observed in HCC cell lines compared with QZG cells. Ectopic expression of miR-125a could inhibit the proliferation, migration and invasion of HCC cells both *in vitro* and *in vivo*. Inversely, down-regulating miR-125a could increase the proliferation, migration and invasion of SMMC-7721 cells. Interestingly, MTT assays showed that the inhibition of HCC cell proliferation by miR-125a after culture for four days was statistically significant; however the end point of the migration and invasion assays was 24 h, indicating that the inhibitory effects on cell migration and invasion were not caused by a reduction of the cell proliferation. Therefore, further experiments are needed to elucidate the mechanism.

MiRNA-125a can translationally arrest the mRNA of the p53 tumor suppressor gene [24], and interfere with the expression of hepatitis B virus surface antigen [25]. Analyses using the miRanda and Pic Tar algorithms indicated that several cancer-associated genes including MMP11, ERBB2, ERBB3, EDN1, VEGF-A, MMP14 and BCL-9L, were potential target genes of miR-125a. Interestingly, over-expression of miR-125a did not significantly repress the expression of ERBB2 or ERBB3, which were demonstrated to be critical for inhibiting the proliferation and metastasis of breast cancer [11]. Instead, luciferase reporter assays confirmed that MMP11 and VEGF-A, but not other genes tested, were targets genes of miR-125a. These findings were further strengthened by the results of qRT-PCR and western blot analyses. Moreover, the co-expression of miR-125a with its targeted genes MMP11 and VEGF-A were also detected in HCC tissues from surgical ablation. The results showed that miR-125a was inversely correlated with both MMP11 and VEGF-A expression in HCC tissues. Based on these data, we suspected that miR-125a could inhibit the proliferation and metastasis of HCC partly through down-regulating MMP11 and VEGF-A.

Previous studies have shown that increased VEGF expression in HCC is associated with a high proliferative index, poor encapsulation of tumors, and venous tumor emboli portal vein thrombosis [26], [27]. SiRNA targeting VEGF can inhibit HCC growth and tumor angiogenesis in vivo [28], and has anti-tumor efficacy for patients with HCC under hypoxic conditions [29]. Our study showed that down-regulation of MMP11 or VEGF-A could rescue the lost of miR-125a function. VEGF expression was significantly correlated with T stage and metastasis in HCC, and MMP11 expression was significantly correlated with the metastasis of HCC. What’s more, our present study showed that lentivirus-mediated MMP11 or VEGF siRNA can rescue the lost of miR-125a function on SMMC-7721 cells, further confirming that miR-125a works through MMP11 or VEGF-A.

In conclusion, our present study showed that miR-125a is frequently down-regulated and inversely correlated with aggressiveness and poor prognosis in HCC. Ectopic expression of miR-125a can inhibit the proliferation, invasion and metastasis of HCC by down-regulating MMP11 and VEGF *in vitro* and *in vivo*, and thus, miR-125a represents a new prognosis marker and a promising approach for HCC treatment.
